# The Impact of SARS-CoV-2 Pandemic on Time to Primary, Secondary Resection and Adjuvant Intravesical Therapy in Patients with High-Risk Non-Muscle Invasive Bladder Cancer: A Retrospective Multi-Institutional Cohort Analysis

**DOI:** 10.3390/cancers13215276

**Published:** 2021-10-21

**Authors:** Matteo Ferro, Francesco Del Giudice, Giuseppe Carrieri, Gian Maria Busetto, Luigi Cormio, Rodolfo Hurle, Roberto Contieri, Davide Arcaniolo, Alessandro Sciarra, Martina Maggi, Francesco Porpiglia, Matteo Manfredi, Cristian Fiori, Alessandro Antonelli, Alessandro Tafuri, Pierluigi Bove, Carlo Terrone, Marco Borghesi, Elisabetta Costantini, Ester Iliano, Emanuele Montanari, Luca Boeri, Giorgio Ivan Russo, Massimo Madonia, Alessandro Tedde, Alessandro Veccia, Claudio Simeone, Giovanni Liguori, Carlo Trombetta, Eugenio Brunocilla, Riccardo Schiavina, Fabrizio Dal Moro, Marco Racioppi, Mihai Dorin Vartolomei, Nicola Longo, Lorenzo Spirito, Felice Crocetto, Francesco Cantiello, Rocco Damiano, Savino M. Di Stasi, Michele Marchioni, Luigi Schips, Paolo Parma, Luca Carmignani, Andrea Conti, Francesco Soria, Paolo Gontero, Biagio Barone, Federico Deho, Emanuele Zaffuto, Rocco Papalia, Roberto M. Scarpa, Vincenzo Pagliarulo, Giuseppe Lucarelli, Pasquale Ditonno, Francesco Maria Gerardo Botticelli, Gennaro Musi, Michele Catellani, Ottavio de Cobelli

**Affiliations:** 1 Department of Urology, IEO European Institute of Oncology, IRCCS, Via Ripamonti 435, 20141 Milan, Italy; FrancescoMariaGerard.Botticelli@ieo.it (F.M.G.B.); Gennaro.Musi@ieo.it (G.M.); michele.catellani@ieo.it (M.C.); ottavio.decobelli@ieo.it (O.d.C.); 2 Department of Oncology and Hematology-Oncology, Università degli Studi di Milano, 20122 Milan, Italy; 3 Department of Urology, Policlinico Umberto I, Sapienza University of Rome, 00161 Rome, Italy; francesco.delgiudice@uniroma1.it (F.D.G.); alessandro.sciarra@uniroma1.it (A.S.); martina.maggi@uniroma1.it (M.M.); 4 Urology and Renal Transplantation Unit, Department of Medical and Surgical Sciences, University of Foggia, 71122 Foggia, Italy; giuseppe.carrieri@unifg.it (G.C.); gianmaria.busetto@unifg.it (G.M.B.); luigi.cormio@unifg.it (L.C.); 5 Department of Urology, Humanitas Research Hospital, IRCCS, 20089 Milan, Italy; rodolfo.hurle@humanitas.it (R.H.); roberto.contieri@humanitas.it (R.C.); 6 Unit of Urology, Luigi Vanvitelli University of Campania, 80138 Naples, Italy; davide.arcaniolo@unicampania.it; 7 Department of Urology, San Luigi Gonzaga Hospital, University of Turin, Orbassano, 10043 Turin, Italy; francesco.porpiglia@unito.it (F.P.); m.manfredi@unito.it (M.M.); cristian.fiori@unito.it (C.F.); 8 Department of Urology, Ospedale Civile Maggiore, Polo Chirurgico Confortini, University of Verona, 37126 Verona, Italy; Alessandro.antonelli@univr.it (A.A.); alessandro.tafuri@univr.it (A.T.); 9 Urology Unit, Azienda Ospedaliera Universitaria Integrata Verona, Piazzale Stefani 1, 37126 Verona, Italy; 10 Department of Urology, San Carlo di Nancy Hospital, Via Aurelia 275, 00165 Rome, Italy; pierluigi.bove@uniroma2.it; 11 Department of Urology, Policlinico San Martino Hospital, University of Genoa, 16132 Genova, Italy; carlo.terrone@unige.it (C.T.); marco.borghesi@unige.it (M.B.); 12 Andrology and Urogynecology Clinic, Santa Maria Terni Hospital, University of Perugia, 05100 Terni, Italy; elisabetta.costantini@unipg.it (E.C.); ester.illiano@unipg.it (E.I.); 13 Urology Unit, Fondazione IRCCS Ca’ Granda Ospedale Maggiore Policlinico, 20122 Milan, Italy; emanuele.montanari@unimi.it (E.M.); luca.boeri@unimi.it (L.B.); 14 Department of Clinical Sciences and Community Health, University of Milan, 20122 Milan, Italy; 15 Department of Urology, University of Catania, 95124 Catania, Italy; giorgioivan.russo@unict.it; 16 Urologic Clinic, Department of Clinical and Experimental Medicine, University of Sassari, 07100 Sassari, Italy; madonia@uniss.it (M.M.); alextedde89@gmail.com (A.T.); 17 Division of Urology, VCU Health System, Richmond, VA 23298, USA; alessandro.veccia@asst-mantova.it; 18 Urology Unit, ASST Spedali Civili Hospital, 25133 Brescia, Italy; 19 Urology Unit, Department of Medical and Surgical Specialties, Radiological Science and Public Health, University of Brescia, 25121 Brescia, Italy; claudio.simeone@unibs.it; 20 Department of Urology, ASUITS, University of Trieste, 34149 Trieste, Italy; giovanni.liguori@asugi.sanita.fvg.it (G.L.); trombcar@units.it (C.T.); 21 Department of Urology, University of Bologna, S-Orsola-Malpighi Hospital, 40138 Bologna, Italy; eugenio.brunocilla@unibo.it (E.B.); riccardo.schiavina3@unibo.it (R.S.); 22 Urology Unit, Department of Surgical, Oncological and Gastroenterological Sciences, University of Padua, 35128 Padua, Italy; fabrizio.dalmoro@unipd.it; 23 Urology Clinic, A. Gemelli Hospital Foundation, Catholic University of the Sacred Heart, IRCCS, 00168 Rome, Italy; marco.racioppi@policlinicogemelli.it; 24 Urology Department, Medical University of Vienna, A-1090 Vienna, Austria; mihai.vartolomei@umfst.ro; 25 IOSUD, Universitatea de Medicina Farmacie Stiinte si Tehnologie “George Emil Palade” din Targu Mures, 540142 Mureș, Romania; 26 Urology Unit, Department of Neurosciences, Reproductive Sciences and Odontostomatology, University of Naples Federico II, 80131 Naples, Italy; nicola.longo@unina.it (N.L.); lorenzo.spirito@unina.it (L.S.); felice.crocetto@unina.it (F.C.); cantiello@unicz.it (F.C.); biagio.barone@unina.it (B.B.); 27 Department of Urology, Magna Graecia University of Catanzaro, 88100 Catanzaro, Italy; damiano@unicz.it; 28 Department of Surgery and Experimental Medicine, Tor Vergata University, 00133 Rome, Italy; dstsnm00@uniroma2.it; 29 Department of Medical, Oral and Biotechnological Sciences, Urology Unit, “SS. Annunziata” Hospital, G. d’Annunzio University of Chieti, 66100 Chieti, Italy; michele.marchioni@unich.it (M.M.); luigi.schips@unich.it (L.S.); 30 Department of Urology, ASL Abruzzo 2, 65017 Chieti, Italy; 31 Urology Unit, Ospedale San Carlo Poma, 46100 Mantova, Italy; paolo.parma@asst-mantova.it; 32 Department of Urology, San Donato Policlinic Hospital, 20094 Milan, Italy; luca.carmignani@unimi.it (L.C.); andrea.conti@grupposandonato.it (A.C.); 33 Department of Surgical Sciences, Division of Urology, San Giovanni Battista Hospital, University of Studies of Torino, 10121 Turin, Italy; francesco.soria@unito.it (F.S.); paolo.gontero@unito.it (P.G.); 34 Unit of Urology, ASST Sette Laghi-Circolo e Fondazione Macchi Hospital, 21100 Varese, Italy; federico.deho@asst-settelaghi.it (F.D.); emanuele.zaffuto@asst-settelaghi.it (E.Z.); 35 Department of Urology, Campus Biomedico University Hospital, 00198 Rome, Italy; rocco.papalia@unicampus.it (R.P.); r.scarpa@unicampus.it (R.M.S.); 36 Urology Unit, Ospedale Vito Fazzi, 73100 Lecce, Italy; vincenzo.pagliarulo@uniba.it; 37 Urology, Andrology and Kidney Transplantation Unit, Department of Emergency and Organ Transplantation, University of Bari, 70124 Bari, Italy; giuseppe.lucarelli@uniba.it (G.L.); pasquale.ditonno@uniba.it (P.D.); 38 Department of Oncology and Hemato-Oncology, University of Milan, 20122 Milan, Italy

**Keywords:** bladder cancer, SARS-CoV-2, intravesical BCG, trans-urethral resection of bladder tumor, Re-TURBT

## Abstract

**Simple Summary:**

The worldwide COVID-19 emergency has had an important impact on healthcare systems with the need to assist infected patients and also treat non-deferrable oncological conditions. In urology, the main concern has been for patients with bladder cancer, the tenth most common malignancy, where the quality and the alacrity of treatment has a clear well-demonstrated impact on the survivor. The aim of our Italian multi-institutional retrospective study was to assess the impact of the COVID-19 outbreak on diagnosis and treatment of non-muscle invasive bladder cancer. We observed a significant delay between diagnosis and surgical treatment, with a lower adherence to the standard therapeutic scheme such as BCG intravesical instillation and urological guidelines. We also recorded a different attitude in treatment depending on the patients’ location in Italy. Further investigation could show the impact of the pandemic on the survival of these patients.

**Abstract:**

Background: To investigate the impact of COVID-19 outbreak on the diagnosis and treatment of non-muscle invasive bladder cancer (NMIBC). Methods: A retrospective analysis was performed using an Italian multi-institutional database of TURBT patients with high-risk urothelial NMIBC between January 2019 and February 2021, followed by Re-TURBT and/or adjuvant intravesical BCG. Results: A total of 2591 patients from 27 institutions with primary TURBT were included. Of these, 1534 (59.2%) and 1056 (40.8%) underwent TURBT before and during the COVID-19 outbreak, respectively. Time between diagnosis and TURBT was significantly longer during the COVID-19 period (65 vs. 52 days, *p* = 0.002). One thousand and sixty-six patients (41.1%) received Re-TURBT, 604 (56.7%) during the pre-COVID-19. The median time to secondary resection was significantly longer during the COVID-19 period (55 vs. 48 days, *p* < 0.0001). A total of 977 patients underwent adjuvant intravesical therapy after primary or secondary resection, with a similar distribution across the two groups (*n* = 453, 86% vs. *n* = 388, 86.2%). However, the proportion of the patients who underwent maintenance significantly differed (79.5% vs. 60.4%, *p* < 0.0001). Conclusions: The COVID-19 pandemic represented an unprecedented challenge to our health system. Our study did not show significant differences in TURBT quality. However, a delay in treatment schedule and disease management was observed. Investigation of the oncological impacts of those differences should be advocated.

## 1. Introduction

The American Cancer Society estimates about 83,730 new diagnoses of bladder cancer (BC) and 17,200 deaths in 2021 [1]. BC is the fourth most common cancer in men, but it is less common in women [2]. About 75% of newly diagnosed BCs are identified as non-muscle invasive (NMIBC) disease, i.e., limited to the mucosa (Ta and carcinoma in situ (CIS)) or to the lamina propria (T1) [3].

The severe acute respiratory syndrome coronavirus 2 (SARS-CoV-2) and the related disease, coronavirus disease 2019 (COVID-19), quickly generated a tragic health emergency in Italy due to the concurrent need to provide assistance to infected patients, and at the same time, to treat all the non-deferrable oncological and benign conditions [4].

The associated reallocation of resources needed to properly assist critically ill COVID-19 patients caused a similar redistribution of the activities of several medical disciplines not primarily involved in the care of COVID-19 patients [5]. Furthermore, the suspension of all outpatient and non-urgent activities, added to the restrictions in the scheduling of non-deferrable procedures, determined a major reorganization of urological activities [6,7,8,9,10].

For these reasons, it was challenging to meet the suggested timescales for NMIBC management [11]. In fact, non-muscle invasive bladder cancer (NMIBC) is an extremely time-sensitive disease due to its pathological characteristics, and prompt diagnosis and therapy are required for better clinical outcomes. Any delay in care concerning both time to diagnosis and time to treatment is associated with a higher pathological stage and a poor prognosis, especially for high-grade (HG) NMIBC [12].

This was the reason why, in 2006, a Canadian consortium of experts proposed a recommended maximum wait time of <14 days in cases of high-risk NMIBC and of <42 days in other types of NMIBC from the onset of symptoms and GP referral [13]. Regarding surgery, Rouprêt et al. also suggested that patients with NMIBC should undergo TURBT in < 1 month as prolonged surgical waiting time has an undeniable impact on the clinical outcomes, quality of life and anxiety of patients [3,14]. Moreover, a residual T1 HG/G3 tumor at Re-TURBT confers a worse prognosis in patients with primary T1 HG/G3 treated with maintenance BCG, and patients are very likely to fail BCG therapy alone [15].

Furthermore, in high-grade tumors, a full dose of BCG therapy lasting 3 years is associated with a reduction in recurrence, but not with a lower progression or a better overall survival; this implies that a shorter treatment is associated with worse outcomes [16].

Taking it all together, the aim of this multicenter study was to investigate the impact of the COVID-19 outbreak on the diagnosis and treatment of NMIBC.

## 2. Materials and Methods

### 2.1. Study Design and Eligibility Criteria

The study was conducted as retrospective and all participating sites provided institutional data sharing agreements prior to the initiation of the trial. Each participant enrolled in the study signed an informed consent before undergoing intravesical BCG therapy according to the European Association of Urology (EAU) [17], Good Clinical Practice (GCP) guidelines [18], ethical principles of the latest version of the Declaration of Helsinki and General Data Protection Regulation (GDPR).

We performed a retrospective analysis of our Italian multi-institutional database of patients who underwent TURBT ± Re-TURBT followed by adjuvant intravesical BCG or MMC for histologically confirmed urothelial high-risk NMIBC between January 2019 and February 2021. The range of the study time was symmetrically distributed in order to obtain a balanced period of enrollment that would allow stratification of our cohort with regard to the pre-COVID-19 vs. COVID-19 period. We set 9 March 2020, as the reference line to define treatments that occurred within the Italian SARS-CoV-2 outbreak.

All the participants’ institutions were grouped into three different geographical areas, according to the Italian macro-regions (Northern, Central and Southern Italy), and were further stratified according to their case volume contribution, which was presented as quartile variations for each center’s enrollment.

Days from diagnosis to primary TURBT, from TURBT to Re-TURBT and from TURBT/Re-TURBT to adjuvant intravesical treatment initiation were collected for the whole cohort and presented together with demographic, clinic-pathological characteristics and all available covariates that could potentially influence the time to treatment during the pre- COVID-19 vs. COVID-19 period.

Patients with primary muscle-invasive disease (MIBC), non-urothelial carcinoma, with incomplete/missing data, or who received treatment for the specific diagnosis of interest later than 1 year after diagnosis, and 6 months following primary resection or Re-TURBT were excluded together with those patients who were treated with non-curative intervention.

### 2.2. Statistical Analysis

Statistical analyses as well as reporting and interpretation of the findings were conducted according to established guidelines and consisted of three analytical steps [19]. First, descriptive statistics were used to summarize the pertinent study information. The association between clinical, demographic, and peri-treatment variables reported as percentages (%), and/or median (IQR) during the pre-COVID-19 and COVID-19 period were tested by Student’s *t*-test or Fisher’s Exact for continuous variables and by the Pearson Chi-squared or Mann–Whitney U test for categorical variables when appropriate.

Second, the univariate effect of the COVID-19 period on time to treatment outcomes was explored by the Kaplan–Meier product-limit method. The log-rank test assessed crude subgroup differences subsequently adjusted for multiple confounders appropriate for the topic of interest.

Third, three separated sets of univariate logistic regression models were developed by testing each potential factor (both dichotomized or continuous variables) influencing the observed median time to TURBT, Re-TURBT and adjuvant intravesical treatment, with significance set at *p* ≤ 0.05. Subsequent specific multivariable stepwise regression models (forward selection) were further generated by selecting those predictive variables that were significant upon univariate analysis, by entering and removing limits set at *p* = 0.05 and *p* = 0.10, respectively. In particular, covariates for each endpoint consisted of center-based, diagnostic-based, tumor-based, and COVID-19 period features as listed below in the respective tables.

Finally, the locally weighted scatter-plot smoother (LOWESS) function was used on the sole sub-group of COVID-19 period patients to graphically depict the predicted probability of a median longer time to intervention according to the three different geographical regions of provenience and according to the single-center volume case quartile distribution.

## 3. Results

### 3.1. Study Cohort Characteristics

According to pre-established criteria, the final cohort who received at least the primary TURBT consisted of *n* = 2591 patients who underwent resection from a total of *n* = 27 academic or non-academic institutions through the whole of Italy. The majority of the enrolling centers were from Northern Italy with *n* = 14 institutions followed by *n* = 5 and *n* = 8 institutions from Southern and Central Italy, respectively, with similar correspondence relative to the regions’ influence in terms of case recruitment. The whole study cohort baseline and first TURBT peri-operative characteristics were divided according to COVID-19 period and are summarized in Table 1. Of these, *n* = 1534 (59.2%) patients underwent primary resection before the COVID-19 outbreak and *n* = 1056 (40.8%) patients during COVID-19. There was only a slight but significant difference between the pre and COVID-19 period in terms of the percentage of recruitment, especially within the Northern institutions (50.7% vs. 45.3%). Out of the whole cohort, the median case volume was 74 (49–109) patients for each center, with a significant difference in terms of patients treated within the pre and COVID-19 period, especially for those among the 4th quartile volume distribution (59.3% vs. 50%).

The diagnostic modality strategies to detect BC were found to be slightly, but significantly different across the COVID-19 period. In particular, there was a minimal trend toward more direct visual inspection of the suspected lesions with more cystoscopies performed (29.1% vs. 34.2%), while the choice of a combined diagnostic strategy was clearly reduced down to only 3% of the sample.

Ultimately, no further significant or clinically relevant differences were identified among the demographic variables, diagnostic tumor features, perioperative characteristics, and histopathological findings.

### 3.2. Time from Diagnosis to Primary TURBT

The time from identification of a bladder lesion to primary resection was significantly longer during the COVID-19 period with a median of 65 (33–84) days vs. 52 (29–75) (*p* = 0.002).

**Figure 1:**
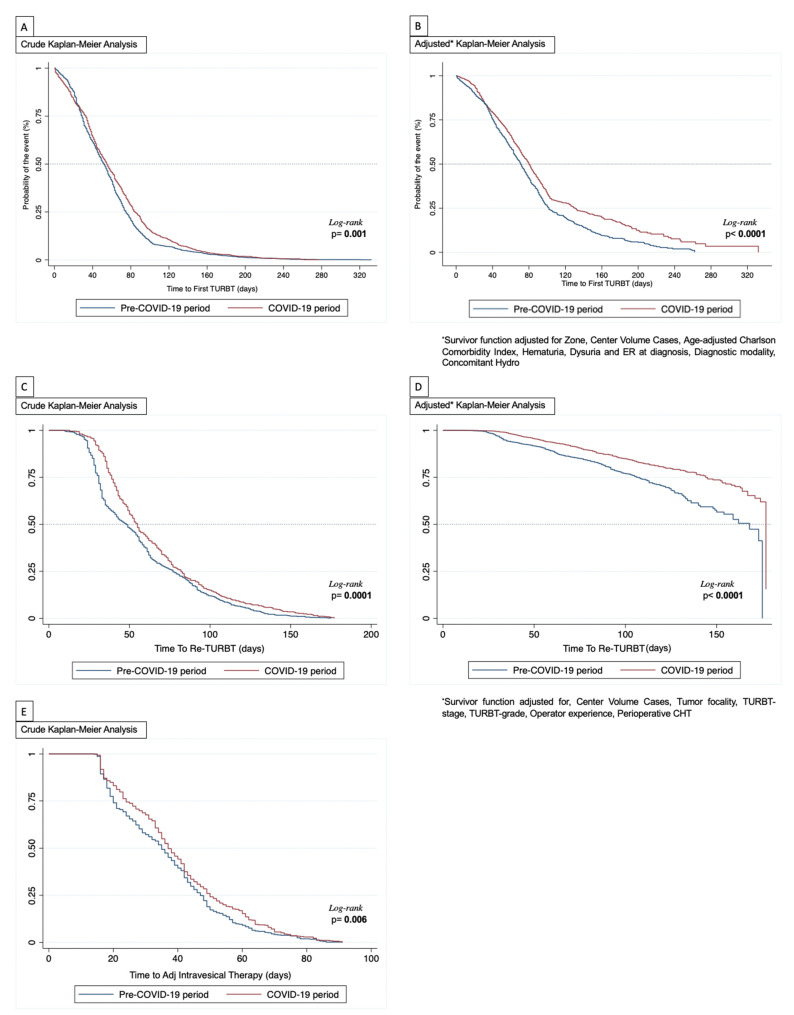
Kaplan–Meier analysis. (**A**) Crude analysis for time to first TURBT; (**B**) Adjusted analysis for time to first TURBT; (**C**) Crude analysis for time to Re-TURBT; (**D**) Adjusted analysis for time to Re-TURBT; (**E**): Crude analysis for time to adjuvant intravesical therapy.

**Figure 2:**
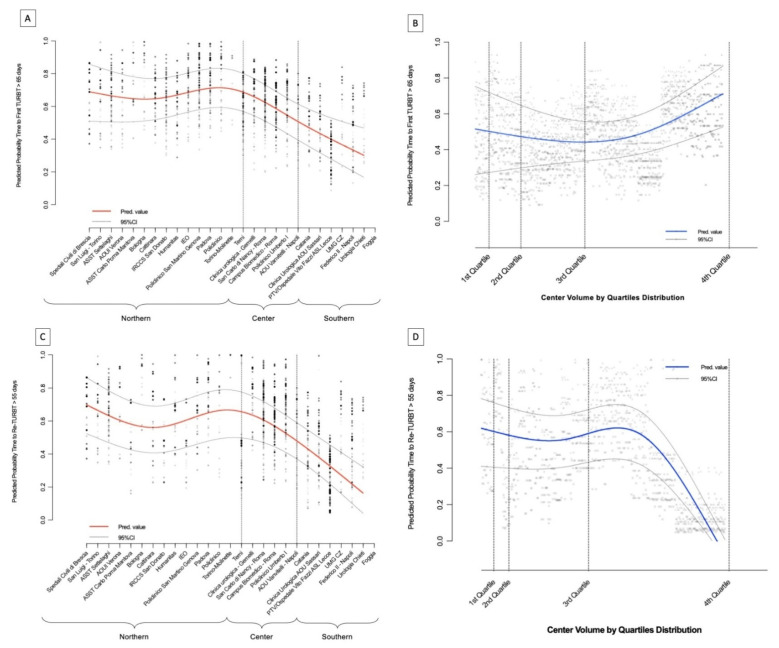
LOWES functions. (**A**) Predicted probability time to first TURB > 65 days among institutions; (**B**) Predicted probability time to first TURB > 65 days among center volume percentiles; (**C**) Predicted probability time to Re-TURB > 55 days among institutions; (**D**) Predicted probability time to Re-TURB > 55 days among center volume percentiles.

Kaplan–Meier analysis showed that the 30-days to TURBT residual function was 72.6% (95%CI: 69.9–74.4) and 76.7% (95%CI: 74.2–79.3) during the pre vs. COVID-19 period, respectively. Similarly, at 60 and 90-days the residuals for those who had not yet undergone TURBT were 41.1% (95%CI: 38.6–43.6), 45.6% (95%CI: 42.6–48.6) and 14.1% (95%CI: 12.3–15.8), 21.3% (95%CI: 18.8–23.8), respectively (log-rank, *p* = 0.001; Figure 1A). The same tendency was observed when the residual function was adjusted for the factors independently influencing the median time to TURBT (Figure 1B). Additionally, multivariable logistic regression analysis showed that a primary resection during the COVID-19 period was an independent predictor for delayed median time to TURBT (OR, 1.26, 95%CI: 1.06–1.51; Table 2). Finally, when analyzing only the last sub-group of patients who underwent TURBT during the COVID-19 period, the LOWESS function depicted an increased predicted probability to receive a primary resection with a median time > 65 days in the Northern centers, while this prediction was linearly reduced for Central and Southern centers (Figure 2A). Interestingly, the probability of a longer time to primary resection was almost exponentially increased among those institutions with a baseline higher case volume (Figure 2B).

### 3.3. Time from TURBT to Secondary Resection (Re-TURBT)

Within the study population, *n* = 1066 (41.1%) received Re-TURBT with *n* = 604 (56.7%) during the pre-COVID-19 and *n* = 462 (43.3%) during COVID-19 period. The median time to secondary resection was significantly longer during the COVID-19 period with a median of 55 (39–82) days vs. 48 (31–77) days, respectively (*p* < 0.0001) (Table 3).

The Kaplan Meier analysis showed that the 30, 60 and 90-days to Re-TURBT residual function were 76% (95%CI: 72.6–79.4) vs. 91.8% (95%CI: 89.3–84.3), 37.4% (95%CI: 36.4–46.3) vs. 43.7% (95%CI: 39.2–48.2) and 17.2% (95%CI: 14.2–20.2) vs. 19.5% (95%CI: 15.9–23.1) during the pre and COVID-19 period, respectively (log-rank, *p* < 0.0001; Figure 1C), even after adjusting for confounders as shown in Figure 1D. Similar to the first TURBT, the multivariable logistic regression analysis showed that the COVID-19 period was an independent predictor for experiencing delayed time to secondary resection (OR: 1.30, 95%CI: 1.05–1.71; Table 4). Of note, as depicted from the LOWESS function only from the COVID-19 months and similarly to what was observed for the primary TURBT analysis, there was a comparable trajectory for the predicted probability of experiencing a median time to Re-TURBT > 55 days among the Northern through to the Southern institutions (Figure 2C). Differently, the probability of having a delayed Re-TURBT was significantly diminished if the surgery was performed in an institution enrolling a high case volume (i.e., 3rd or 4th quartile volume distribution; Figure 2D).

### 3.4. Time from TURBT/Re-TURBT to Adjuvant Intravesical Therapy

The sample who underwent adjuvant intravesical therapy was limited to *n* = 977 patients, accounting for *n* = 527 (53.9%) and *n* = 450 (46.1%) during the pre and COVID-19 period. As expected, the vast majority of the patients who received adjuvant BCG were equally distributed across the non-COVID-19 or COVID-19 period (*n* = 453, 86% vs. *n* = 388, 86.2%, respectively; Table 5). In addition, the proportion of patients who underwent induction plus a maintenance course during the COVID-19 period was reduced when compared to the non-COVID-19 period (79.5% vs. 60.4%, *p* < 0.0001), while among patients in a maintenance course only, the SWOG schedule was longer than 12 months (24.9% vs. 4.2%, *p* < 0.0001).

Kaplan–Meier analysis showed that the 30 and 60-days from last TURBT to adjuvant intravesical therapy were 57.1% (95%CI: 52.9–61.3) vs. 67.6% (95%CI: 63.2–71.9), and 9.1% (95%CI: 6.7–11.6) vs. 15.3% (95%CI: 12–18.7) during the pre and COVID-19 period, respectively (log-rank, *p* = 0.006; Figure 1E). Although the COVID-19 period was a risk factor upon univariate analysis (OR: 1.25, 95%CI: 0.97–1.61), multivariable logistic regression analysis showed it was not independently associated with delayed time to the beginning of adjuvant intravesical therapy (OR: 1.11, 95%CI: 0.84–1.38; Table 6).

## 4. Discussion

As shown in our study, the COVID-19 outbreak led to a delay in surgical therapy (TURBT and Re-TURBT). To mitigate the potential impact of procedure deferral, the EAU proposed additional guidelines to help urologists in their activity [20,21]. The EAU categorized diagnoses of NMIBC into four priority groups according to clinical harm: low priority patients, who should be postponed by 6 months (small papillary recurrences < 1 cm and/or history of Ta/1 low-grade BC); intermediate (BC > 1cm) and high priority patients (high-risk BC or macroscopic hematuria) who should be not postponed beyond 3–4 months and 6 weeks, respectively. In addition, immediate radical cystectomy has been suggested in case of high-risk NMIBC or BCG failure while, reasonably, emergencies should be diagnosed and treated as soon as possible (e.g., macroscopic hematuria with clot retention) [20].

Our study showed that the diagnostic strategies used to detect BCs changed during the COVID-19 period. More specifically, a minimal trend toward more direct visual inspection of the suspected lesions was observed, with more cystoscopies performed (29.1% vs. 34.2%). We can hypothetically explain this trend by the lower outpatient activity (such as the US) determined by resource optimization for the pandemic, and also by with limited use of urinary biomarkers due to their accuracy, availability and high costs. Moreover, we found a reduced use of combined diagnostic strategy, down to only 3% of the sample.

Secondly, the time to treatment during the COVID-19 period was significantly prolonged when compared to times before the pandemic (65 vs. 52 days). In addition, the decreased activity of general practitioners as well as the residents in small or medium-sized cities could have further impacted those delays [22]. The length of the surgical wait time is of crucial importance in BC and patients should undergo a TURBT within 30 days. A delay of over 68 days in this procedure worsens the overall survival at 1, 3 and 5 years as reported by Wallace et al. and therefore, we expect inauspicious outcomes for the sample of patients of the COVID-19 period in the future [23].

Interestingly, our study shows how the probability of a longer time to primary resection was almost exponentially increased among those institutions with a higher case volume baseline, which tends to coincide with the Northern centers. This indicates more difficulties in hospital organization due to the higher number of hospitalized COVID-19 cases. In fact, the reallocation of medical personnel to new COVID-19 wards and the associated reduction in active personnel due to the COVID-19 infection, produced a dramatic change in routine clinical and surgical practice, as already demonstrated by Naspro et al. [24,25].

Similarly, time to Re-TURBT was prolonged during the COVID-19 pandemic (55 vs. 48 days), even though EAU guidelines for NMIBC suggest the second resection 2–6 weeks after the initial TURBT. In contrast with time to first treatment, high volume centers had a shorter time to Re-TURBT when compared to institutions with a smaller volume of cases, which were located in Central and Southern Italy. It is clear that there are many socioeconomic differences across Italy and this results in better organization in the Northern part where oncological hubs were created for better management of patients with BC. Oncologic hub hospitals must fulfill specific requirements, which include: the role as a referral center with high surgical volume and experience; low risk for complications and prolonged hospitalization; the ability to treat oncologic patients in dedicated spaces in order to preserve immunosuppressed subjects from possible COVID-19 infections; the presence of sustainable resources for infrastructural, medical and paramedical necessities aimed to reduce the deferral of cancer patients during the COVID-19 pandemic [26].

Regarding BCG therapy, our results showed that the percentage of patients treated with immunotherapy during the COVID-19 period was comparable to the pre-COVID-19 era (86.2 vs. 86%). We noticed a delay in the 30- and 60- days from last TURBT to adjuvant intravesical BCG administration across the two periods, 57.1% vs. 67.6% and 9.1% vs. 15.3%, respectively.

In addition, our study showed a reduced proportion of patients who underwent BCG therapy (induction + maintenance) after surgery during the COVID-19 period (60.4%) with more difficulty in following the SWOG schedule longer than 12 months. Patients involved in a BCG scheme in the years before the COVID-19 pandemic had more difficulties in maintaining the immunotherapy. As we have learnt from BCG shortages in past, the difference between 3 years maintenance compared to 1 year of maintenance was significant regarding recurrence rate, although no effect on progression or death has been reported [27].

A delay in cancer treatment and disturbances in cancer care during the COVID-19 period was also reported by Schimdt et al., who outlined a significant disruption to cancer care during the pandemic and a decrease in outpatient visits at tertiary institutions in New York and Boston [28]. Similar findings were also reported in case of patients diagnosed with oral squamous cell carcinoma, with a treatment delay in 2020 of 45 days compared to 35 days in the 2010–2019 period (*p* = 0.004) [29]. A systematic review concluded that patients and caregivers experienced delays in screening, treatment and care of cancer during the COVID-19 pandemic [30].

## 5. Limitations of the Study

To our knowledge, this is the first study to test the impact of the SARS-CoV-2 pandemic on the management of HG-NMIBC, in particular on time to treatment, time to Re-TURBT and BCG administration. Some limits should be taken into consideration. Firstly, our study is based on a retrospective analysis of data, which implies the impossibility of predicting the impact of the pandemic on the clinical outcomes of our sample of patients. To better understand the role of the lack of NMIBC management, a long-term follow-up of the same patients should be conducted in the next few years. Secondly, the distribution of the sample of patients was not uniform across Italy, because of the difficulties in collecting data from those non-academic institutions overwhelmed by COVID-19 emergencies.

## 6. Future Perspectives

The COVID-19 pandemic not only determined a redistribution of the activities of several medical disciplines but also had a clear impact on oncological patient therapies, as we demonstrated for HG-NMIBC. The actual impact of SARS-CoV-2 on clinical outcomes is still to be understood. To reduce the delay in BC management several diagnostic strategies can be implemented. Firstly, we recommend better adherence to the guidelines in order to obtain better stratification of patients with HG-NMIBC [31]. Secondly, the Vesical Imaging-Reporting and Data System (VI-RADS) may offer a reliable first-step diagnostic tool in identifying and prioritizing patients who would benefit from immediate intervention [32]. Thirdly, the expansion of the role of urinary biomarkers in diagnostic and surveillance pathways could be a feasible strategy to solve the waiting times for cystoscopies [33,34].

## 7. Conclusions

The COVID-19 pandemic represented a novel and groundbreaking challenge to our health system, and also heavily influenced the training and education of urology residents. According to our study, although TURBT quality was not significantly affected by the COVID-19 pandemic, a delay in treatment schedules and disease management was observed. Further, the oncological impact should be investigated, in order to assess the whole impact of the COVID-19 pandemic on the outcomes of patients with NMIBC.

## Figures and Tables

**Table 1:** Descriptive characteristics of the study cohort. In bold, value < 0.05.

Primary TURBT Demographic and Clinic-Pathological Features					
Variables	Pre-Covid-19 Period	%	Covid-19 Period	%	*p-*Value
**Sample size,** *n* (%)	1535	59.2	1056	40.8	
**Regions of provenience,** *n* (%)					**<0.0001**
Northern Italy	778	50.7	478	45.3	
Central Italy	380	24.8	351	33.2	
Southern Italy	377	24.6	227	21.5	
**Center volume case,** quartiles					**<0.0001**
1st quartile	134	8.7	72	6.8	
2nd quartile	211	13.7	189	17.9	
3rd quartile	280	18.2	267	25.3	
4th quartile	910	59.3	528	50.0	
**Median age**, years (IQR)	74 (68–81)		74 (66–81)		0.247
**Gender**, *n* (%)					0.429
Male	1222	79.6	854	80.9	
Female	313	20.4	202	19.1	
**Smoking status**, *n* (%)					**0.001**
Never	629	41.0	450	42.6	
Active	860	56.0	599	56.7	
Former	46	3.0	7	0.7	
**ACCI score**, *n* (%)					**0.011**
0–2	504	32.8	297	28.1	
≥3	1031	67.2	759	71.9	
**Hematuria at diagnosis**, *n* (%)					0.539
No	509	33.2	338	32.0	
Yes	1026	66.8	718	68.0	
**Dysuria at diagnosis,** *n* (%)					**0.001**
No	1132	73.7	837	79.3	
Yes	403	26.3	219	20.7	
**ER access at diagnosis**, *n* (%)					0.086
No	1310	85.3	874	82.8	
Yes	225	14.7	182	17.2	
**Diagnosis modality**, *n* (%)					**<0.0001**
Ultrasound	781	50.9	517	49.0	
CT scan	172	11.2	143	13.5	
Cystoscopy	446	29.1	361	34.2	
All combined	136	8.9	35	3.3	
**Urinary cytology**, *n* (%)					0.432
Not performed	925	60.3	663	62.8	
Negative for TCC	239	15.6	154	14.6	
Positive for TCC	371	24.2	239	22.6	
**Diagnostic tumor findings**					
**Tumor focality**, *n* (%)					0.478
Unifocal	885	57.7	594	56.3	
Multifocal	650	42.3	462	43.8	
**Ureteral orifice involvement**, *n* (%)					**0.034**
No	1463	95.3	1024	97.0	
Yes	72	4.7	32	3.0	
**Concomitant Hydronephrosis**, *n* (%)					0.359
No	1411	91.9	981	92.9	
Yes	124	8.1	75	7.1	
**Concomitant UTUC**, *n* (%)					**0.003**
No	1463	95.3	1030	97.5	
Yes	72	4.7	26	2.5	
**Perioperative characteristics**					
**Median time from diagnosis to TURBT,** days (IQR)	52 (29–75)		65 (33–84)		**0.002**
**Tumor size**, *n* (%)					0.469
<3 cm	1136	74.0	768	72.7	
≥3 cm	399	26.0	288	27.3	
**Tumor T stage**, *n* (%)					0.105
T0/Tx	48	3.1	40	3.8	
Ta	625	40.7	434	41.1	
T1	776	50.6	516	48.9	
≥T2	35	2.3	40	3.8	
Tis	51	3.3	26	2.5	
**Detrusor in the specimen**, *n* (%)					0.136
Present	1162	75.7	826	78.2	
Absent	373	24.3	230	21.8	
**Tumor histology**, *n* (%)					0.563
TCC	1476	96.2	1020	96.6	
Other	59	3.8	36	3.4	
**CIS**, *n* (%)					0.376
Absent	1380	89.9	964	91.3	
Pure CIS	51	3.3	26	2.5	
Concomitant CIS	104	6.8	66	6.3	
**LVI**, *n* (%)					0.058
Absent	1465	95.4	990	93.8	
Present	70	4.6	66	6.3	
**Operator experience**, *n* (%)					0.347
≥100 TURBTs	1251	81.5	845	80.0	
<100 TURBTs	284	18.5	211	20.0	
**Perioperative intravesical CHT**, *n* (%)					**0.007**
None	1485	96.7	1008	95.5	
Mitomycin-C	26	1.7	37	3.5	
Epirubicin	24	1.6	11	1.0	

**Table 2:** Descriptive characteristics of Re-TURBT cohort. In bold, value < 0.05.

Re-TURBT Demographic and Clinic-Pathological Features					
Variables	Pre-COVID-19 Period	%	COVID-19 Period	%	*p* Value
**Sample size**, *n* (%)	604	56.7	462	43.3	
**Regions of provenience**, *n* (%)					**0.015**
Northern Italy	283	46.9	223	48.3	
Central Italy	76	12.6	83	18.0	
Southern Italy	245	40.6	156	33.8	
**Center case volume**, quartiles					**<0.0001**
1st quartile	65	10.8	25	5.4	
2nd quartile	52	8.6	40	8.7	
3rd quartile	85	14.1	146	31.6	
4th quartile	402	66.6	251	54.3	
**Median age**, years (IQR)	74 (65–80)		74 (67–80)		0.332
**Gender**, *n* (%)					0.621
Male	495	82.0	384	83.1	
Female	109	18.0	78	16.9	
**ACCI score**, *n* (%)					0.567
**Perioperative features**, *n* (%)					
**Median time to Re-TURBT**, days (IQR)	48 (31–77)		55 (39–82)		**<0.0001**
**Re-TURBT T stage**, *n* (%)					0.714
T0/Tx	352	58.3	258	55.8	
Ta	103	17.1	81	17.5	
T1	86	14.2	76	16.5	
≥T2	23	3.8	13	2.8	
Tis	40	6.6	34	7.4	
**Tumor Grade** (WHO 2004), *n* (%)					0.100
Negative	354	58.6	258	55.8	
LG	56	9.3	31	6.7	
HG	194	32.1	173	37.4	
**CIS**, *n* (%)					0.399
Not applicable	515	85.3	381	82.5	
Pure CIS	49	8.1	48	10.4	
Concomitant CIS	40	6.6	33	7.1	
**Operator experience**, *n* (%)					0.264
≥100 TURBTs	134	22.2	116	25.1	
<100 TURBTs	470	77.8	346	74.9	

**Table 3:** Descriptive characteristics of adjuvant intravesical therapy cohort. In bold, value < 0.05.

Adjuvant Intravesical Therapy Demographic and Treatment Schedule					
Variables	Pre-COVID-19 Period	%	COVID-19 Period	%	*p* Value
**Sample size**, *n* (%)	527	53.9	450	46.1	
**Regions of provenience**, *n* (%)					**<0.0001**
Northern Italy	298	56.5	220	48.9	
Central Italy	44	8.3	113	25.1	
Southern Italy	185	35.1	117	26.0	
**Center case volume**, quartiles					**<0.0001**
1st quartile	34	6.5	37	8.2	
2nd quartile	33	6.3	44	9.8	
3rd quartile	46	8.7	124	27.6	
4th quartile	414	78.6	245	54.4	
**Median age**, years (IQR)	74 (68–80)		73 (65–79)		**0.038**
**Gender**, *n* (%)					0.209
Male	429	81.4	380	84.4	
Female	98	18.6	70	15.6	
**ACCI score**, *n* (%)					0.276
0–2					
≥3	382	72.5	340	75.6	
**Median time to Adj Intravesical Therapy**, days (IQR)	35 (20–47)		37 (24–50)		
**Intravesical Drug**, *n* (%)					0.905
Mitomycin-C	74	14.0	62	13.8	
BCG	453	86.0	388	86.2	
**Intravesical Adj schedule**, *n* (%)					**<0.0001**
Only Induction	94	17.8	143	31.8	
Induction + Maintenance	419	79.5	272	60.4	
**SWOG BCG maintenance**, *n* (%)					**<0.0001**
3 months	27	5.1	53	11.8	
6 months	49	9.3	44	9.8	
12 months	131	24.9	19	4.2	
>12 months	139	26.4	65	14.4	

**Table 4:** Univariable and multivariable logistic regression analysis for delayed time to secondary resection. In bold, value < 0.05.

Subgroups and/or Continuous Variables		Univariate Analysis		Multivariate Analysis	
		HR (95%CI)	*p* Value	HR (95%CI)	*p* Value
**Region of provenience**	**Northern Italy**	Ref	--		
	Central Italy	1.42 (0.87–2.37)	0.63		
	Southern Italy	0.97 (0.66–1.85)	0.54		
**Center volume case**	1st quartile	Ref	--		
	2nd quartile	0.70 (0.46–1.06)	0.09		
	3rd quartile	1.05 (0.75–1.49)	0.77		
	4th quartile	0.90 (0.65–1.24)	0.53		
**Age**, years	Continuous	1.01 (0.98–1.02)	0.06		
**Age**, years	<70	Ref	--		
	≥70	1.04 (0.88–1.23)	0.66		
**Gender**	Male	Ref	--		
	Female	1.01 (0.83–1.22)	0.94		
**ACCI**, score	0–2	Ref	--	Ref	--
	≥3	2.13 (1.72–2.56)	**<0.0001**	1.80 (1.44–2.26)	**< 0.0001**
**Hematuria at diagnosis**	No	Ref	--	Ref	--
	Yes	0.66 (0.56–0.78)	**<0.0001**	0.80 (0.66–0.97)	**0.023**
**Dysuria at diagnosis**	No	Ref	--	Ref	--
	Yes	0.75 (0.63–0.90)	**0.002**	0.87 (0.71–1.06)	0.16
**ER access at diagnosis**	No	Ref	--	Ref	--
	Yes	0.68 (0.55–0.84)	**0.001**	0.76 (0.59–0.97)	**0.029**
**Diagnosis modality**	Ultrasound	Ref	--	Ref	--
	CT scan	1.38 (1.08–1.76)	**0.011**	1.47 (0.78–1.94)	0.19
	Cystoscopy	1.27 (1.07–1.52)	**0.008**	1.33 (0.66–1.62)	0.26
	All combined	2.51 (1.79–3.53)	**< 0.0001**	1.42 (0.74–2.73)	0.292
**Urinary cytology**	Not performed	Ref	--		
	Negative for TCC	0.84 (0.67–1.04)	0.112	1.28 (0.61–1.56)	0.21
	Positive for TCC	0.49 (0.41–0.60)	**<0.0001**	0.55 (0.44–0.68)	**< 0.0001**
**Tumor focality**	Unifocal	Ref	--		
	Multifocal	0.93 (0.80–1.09)	0.37		
**Ureteral orifice involvement**	No	Ref	--		
	Yes	1.19 (0.80–1.77)	0.38		
**Concomitant Hydronephrosis**	No	Ref	--	Ref	--
	Yes	0.56 (0.42–0.76)	**0.001**	0.69 (0.49–1.96)	0.27
**Concomitant UTUC**	No	Ref	--		
	Yes	0.79 (0.52–1.18)	0.24		
**TURBT period**	Pre-COVID-19	Ref	--	Ref	--
	COVID-19	1.32 (1.11–1.62)	**0.032**	1.26 (1.06–1.51)	**0.01**

**Table 5:** Univariable and multivariable logistic regression analysis for delayed time to adjuvant intravesical therapy (induction). In bold, value < 0.05.

Subgroups and/or Continuous Variables		Univariate Analysis		Multivariate Analysis	
		HR (95%CI)	*p* Value	HR (95%CI)	*p* Value
**Region of provenience**	**Northern Italy**	**Ref**	--		
	Central Italy	1.25 (0.86–1.83)	0.25		
	Southern Italy	0.49 (0.11–2.19)	0.59		
**Center volume case**, quartiles	1st quartile	Ref	--	Ref	--
	2nd quartile	1.43 (0.79–2.61)	0.24	1.19 (0.61–2.11)	0.34
	3rd quartile	0.49 (0.29–0.81)	**0.006**	0.58 (0.39–1.06)	0.24
	4th quartile	0.47 (0.30–0.74)	**0.001**	0.64 (0.45–0.89)	**0.0013**
**ACCI**, score	0–2	Ref	--		
	≥3	1.57 (2.23–1.11)	**0.001**		
**Tumor focality**, *n*	Unifocal	Ref	--	Ref	--
	Multifocal	0.73 (0.57–0.93)	**0.01**	0.75 (0.58–0.99)	**0.039**
**Tumor size**, cm	<3 cm	Ref	--		
	≥3 cm	1.26 (0.96–1.66)	0.1		
**Tumor stage** TNM	Ta	Ref	--	Ref	--
	T1	0.55 (0.42–0.72)	**<0.0001**	0.69 (0.51–0.93)	**0.017**
	Tis	1.66 (0.67–4.13)	0.273		
**Tumor Grade**, WHO 2004	LG	Ref	--	Ref	--
	HG	0.22 (0.12–0.40)	**<0.0001**	0.25 (0.10–0.62)	**<0.0001**
**Detrusor in the specimen**	No	Ref	--		
	Yes	0.62 (0.46–0.84)	**0.002**		
**Tumor histology**	TCC	Ref	--		
	Other	1.16 (0.59–2.30)	0.67		
**Concomitant CIS**	No	Ref	--	Ref	--
	Yes	0.55 (0.37–0.83)	**0.005**	0.71 (0.46–1.09)	0.12
**LVI**	No	Ref	--		
	Yes	1.63 (0.92–2.90)	0.1		
**Operator experience**	≥100 TURBTs	Ref	--	Ref	--
	<100 TURBTs	1.63 (1.23–2.18)	**0.001**	1.42 (1.04–1.95)	**0.028**
**Perioperative CHT**	No	Ref	--	Ref	--
	Yes	3.36 (1.22–9.23)	**0.019**	4.77 (1.57–14.50)	**0.006**
**Concomitant Hydronephrosis**	No	Ref	--		
	Yes	0.98 (0.64–1.52)	0.94		
**Concomitant UTUC**	No	Ref	--		
	Yes	1.21 (0.70–2.09)	0.5		
**Re-TURBT period**	Pre-COVID-19	Ref	--	Ref	--
	COVID-19	1.32 (1.03–1.68)	**0.026**	1.30 (1.05–1.71)	**0.036**

**Table 6:** Univariable and multivariable logistic regression analysis for delayed time to adjuvant intravesical therapy (maintenance). In bold, value < 0.05.

Subgroups and/or Continuous Variables		Univariate Analysis		Multivariate Analysis	
		HR (95%CI)	*p* Value	HR (95%CI)	*p* Value
**Region of provenience**	Northern Italy	Ref	--		
	Central Italy	0.96 (0.66–1.41)	0.85		
	Southern Italy	1.16 (0.55–1.28)	0.72		
**Center volume case**, quartiles	1st quartile	Ref	--	Ref	--
	2nd quartile	0.71 (0.37–1.36)	0.31	0.92 (0.42–1.99)	0.83
	3rd quartile	0.60 (0.30–0.92)	**0.034**	1.03 (0.52–2.06)	0.93
	4th quartile	0.30 (0.17–1.54)	**<0.0001**	0.51 (0.28–0.94)	**0.03**
**ACCI**, score	0–2	Ref	--		
	≥3	0.70 (0.49–1.02)	0.063		
**Adjuvant Intravesical Drug**	Mitomycin-C	Ref	--	Ref	--
	BCG	0.25 (0.16–0.38)	**<0.0001**	0.37 (0.23–0.59)	**<0.0001**
**Tumor stage at TURBT/Re-TUR**	Ta	0.88 (0.55–1.41)	0.60		
	T1	1.62 (1.00–2.63)	0.05		
	Tis	1.60 (0.90–2.84)	0.11		
**Tumor Grade**, WHO 2004	LG	0.92 (0.49–1.74)	0.79		
	HG	1.43 (1.00–2.04)	0.05		
**Concomitant CIS at TURBT/Re-TUR**	No	Ref	--		
	Yes	1.23 (0.57–2.62)	0.60		
**Adjuvant Intravesical period**	Pre-COVID-19	Ref	--	Ref	--
	COVID-19	1.25 (0.97–1.61)	**0.008**	1.11 (0.84–1.38)	0.35

## Data Availability

The data presented in this study are available on request from the corresponding author.
